# Gut microbiota and metabolites as potential modulators of cognitive impairment after aneurysmal subarachnoid hemorrhage: a hypothesis-driven review

**DOI:** 10.3389/fmicb.2026.1841330

**Published:** 2026-08-04

**Authors:** Wentian Zhang, Miaomiao Chen, Tingyu Guo, Hongqin Wang, Ning Ma

**Affiliations:** 1 The First Clinical School of Medicine, Shanxi Medical University, Taiyuan, China; 2 Department of Neurosurgery, Cardiovascular Hospital Affiliated to Shanxi Medical University, Taiyuan, China; 3 Shanxi Key Laboratory of Heart Failure Precision Medicine, Taiyuan, China; 4 Department of Neurosurgery, The First Hospital of Shanxi Medical University, Taiyuan, China; 5 Academy of Medical Sciences, Shanxi Medical University, Taiyuan, China; 6 School of Public Health, Shanxi Medical University, Taiyuan, China

**Keywords:** aneurysmal subarachnoid hemorrhage, cognitive impairment, gut microbiota, mechanisms, metabolites, therapeutic strategies

## Abstract

Cognitive impairment is a major sequela following aneurysmal subarachnoid hemorrhage (aSAH), severely influencing patients’ long-term quality of life. Its mechanism of occurrence is complex and has not been fully clarified to date. In recent years, the role of the gut microbiota in neurological diseases has gradually been revealed, providing a compelling theoretical framework for exploring the gut-brain axis in aSAH-induced cognitive impairment. Recognizing the limitations of current cross-sectional evidence, this review explicitly aims to propose testable hypotheses rather than establish definitive causation. In this study, we propose the novel “gut-derived secondary hit” hypothesis, aiming to explore how secondary gut microbiota dysbiosis, triggered by primary central nervous system injury following aSAH, may act as a potential modulator contributing to long-term cognitive impairment through altered shifts in the metabolite profile. In this review, we outline the clinical manifestations and pathological mechanisms of aSAH-induced cognitive impairment, as well as the observed shifts in gut microbial composition. We further deeply explore the complex pathways through which the gut microbiome and its derived metabolites may be associated with cognitive impairment. In addition, incorporating recent advances, we discuss prospective microbiome-targeted therapeutic strategies, providing a translational perspective for future mechanistic studies and the clinical prevention and treatment of aSAH-induced cognitive impairment.

## 1 Introduction

Subarachnoid hemorrhage (SAH) is a severe subtype of stroke. Spontaneous rupture of an intracranial aneurysm constitutes its primary cause, accounting for approximately 80 to 85% of cases (Maher et al., 2020). aSAH not only leads to high morbidity and mortality but also imposes a profound burden on global healthcare systems. A comprehensive meta-analysis involving 75 studies from 32 countries showed that the annual incidence of spontaneous SAH worldwide is 8 cases per 100,000 people, with an overall declining trend (Etminan et al., 2019). In recent years, with the development of microsurgical techniques and endovascular treatment technologies, the mortality and disability rates of aSAH have gradually decreased. Notably, even among those who successfully survive the acute phase, a striking 27 to 44% of aSAH patients develop persistent cognitive impairment (Wong et al., 2016). These deficits involve multiple cognitive domains, including memory, information processing speed, executive function, and social cognition—severely disrupting patients’ activities of daily living and social functions (Buunk et al., 2016; Burke et al., 2018). Therefore, exploring the mechanisms of the occurrence and development of cognitive impairment in aSAH patients, finding safe and effective targets, and developing preventive treatment strategies are of great significance for the clinical prevention and treatment of aSAH-induced cognitive impairment.

In recent years, with the continuous deepening of understanding of the gut-brain axis, the role of the gut microbiota in neurological diseases has attracted much attention. Accumulating evidence highlights a significant association between gut dysbiosis and many cognitive disorders (Frileux et al., 2024). However, it is imperative to recognize that aSAH is not a generic brain injury. Its pathophysiology is governed by two temporally distinct phases, early brain injury (EBI) and delayed cerebral ischemia (DCI), and is uniquely associated with the profound neurotoxicity of subarachnoid blood accumulation and its degradation products such as heme and free iron. While the majority of current research predominantly focuses on exploring the role of the gut microbiota in the formation and rupture of intracranial aneurysms, its specific involvement in these distinct pathological windows of long-term cognitive impairment following aSAH remains a critical knowledge gap. Therefore, this article aims to bridge this void by proposing a novel “gut-derived secondary hit” hypothesis tailored exclusively to the aSAH microenvironment. We systematically delineate how the intense systemic stress of EBI may trigger initial gut dysbiosis, and how subsequent global shifts in the microbial metabolite profile are hypothesized to synergistically interact with aSAH-specific pathological events. By exploring how the depletion of neuroprotective mediators and the influx of neurotoxins might amplify microglial hyperactivation, heme-driven oxidative stress, and blood–brain barrier disruption, we illustrate how the gut acts as a potential modulator that may be associated with DCI and long-term cognitive decline. Furthermore, we have summarized intervention strategies targeting the gut microbiome, providing novel insights into improving the long-term clinical prognosis for patients with aSAH.

To ensure a comprehensive and rigorous synthesis of the current evidence, and to enhance the reproducibility of this review, we conducted a systematic literature search. Primary databases, including PubMed, ScienceDirect, and Web of Science, were queried for articles published between January 2010 and May 2026. The primary search strategy utilized Boolean logic to combine keywords, formatted primarily as: (“Subarachnoid Hemorrhage” OR “aSAH”) AND (“Cognitive Impairment” OR “Cognition”) AND (“Gut Microbiota” OR “Microbiome” OR “Metabolites”). Articles were included if they were peer-reviewed English-language publications encompassing clinical observational studies, preclinical models specific to the aSAH microenvironment, or analogous neurological and neuroinflammatory disease models strictly utilized for mechanistic extrapolation. Case reports, unverified preprints, and abstracts without full texts were excluded. Furthermore, to deeply elucidate multifaceted biological mechanisms, supplementary targeted searches were independently conducted using specific mechanistic keywords (e.g., “Neuroinflammation,” “Neuroendocrine,” “Vagus Nerve,” “Oxidative Stress,” “Apoptosis,” and “Blood–Brain Barrier”). Notably, while the systematic search focused on recent literature (2010–2026) to reflect the latest advances in the gut-brain axis, a few seminal historical studies published prior to 2010 (e.g., foundational investigations into blood–brain barrier disruption) were meticulously hand-searched and included to establish classic pathophysiological concepts. By critically evaluating these primary and supplementary studies, this review aims to provide a robust methodological foundation for the proposed hypotheses.

## 2 Cognitive impairment following aSAH

Cognition represents the complex integration of sensory input, central processing, and psychological internalization, culminating in the acquisition and application of knowledge (Millan et al., 2012; Aging Biomarker Consortium et al., 2023). This process encompasses multiple dimensions such as memory, language, visuospatial perception, executive function, calculation, comprehension, and judgment (Aging Biomarker Consortium et al., 2023). Cognitive impairment refers to the impairment or decline in one or more aspects of these cognitive functions. Studies have shown that 27 to 44% of patients with aSAH will develop cognitive impairment, which significantly affects their normal work and daily life (Wong et al., 2016). Pathologically, the extravasation of blood into the subarachnoid space results in diffuse brain injury, which may affect multiple cognitive domains, including memory, information processing speed, executive function, and social cognition (Buunk et al., 2016; Burke et al., 2018).

Executive dysfunction represents a common cognitive problem after aSAH, involving abilities such as planning, organization, problem-solving, and task-switching. Pivotal work by Buunk et al. (2016) demonstrated that, compared to healthy controls, aSAH patients exhibit broad deficits across memory, processing speed, and attention. Notably, their higher-order frontal lobe functions (especially executive function) exhibited obvious deficits. Patients may show difficulties in making decisions, inability to effectively manage time and complete complex tasks, leading to a marked decline in quality of life. Furthermore, memory deficits are also one of the most common manifestations of cognitive impairment, hindering both the acquisition of new information and the retrieval of past events. Burke et al. (2018) elucidated that cognitive impairment in aSAH is not limited to isolated executive or attentional deficits but also secondarily affects memory encoding and other non-executive functions.

Furthermore, many patients experience persistent difficulties engaging in leisure activities and social interactions after aSAH (Buunk et al., 2015). A longitudinal study has revealed the long-term trajectory of social cognitive and behavioral issues in aSAH, and clearly pointed out that social cognitive impairments are persistent and refractory, exhibiting limited potential for recovery (Jorna et al., 2025). These cognitive impairments not only affect the patients’ personal lives but also impose additional burdens on families and society. Therefore, understanding the mechanism of the occurrence and development of cognitive impairments in aSAH patients is of crucial importance.

The pathophysiological mechanism of cognitive impairment after aSAH is inherently complex and multifaceted. Crucially, to understand the role of the gut-brain axis in aSAH, it must be delineated across two temporally distinct phases: EBI and DCI, each corresponding to different mechanistic layers of microbiota-gut-brain interactions. Within the initial 72 h following an aSAH event (the EBI phase), the increase in intracranial pressure leads to reduced cerebral perfusion, global cerebral ischemia, accompanied by disruption of the blood–brain barrier, global cerebral edema, and toxic effects of cerebrospinal fluid in the subarachnoid space, which are important factors for the occurrence of EBI (Maher et al., 2020; Thilak et al., 2024). This causes primary damage to the hippocampus and other limbic systems as well as the cerebral cortex. This intense acute physiological stress and neuroendocrine storm act as the primary triggers, initiating rapid structural shifts and dysbiosis within the gut microbiota. Furthermore, DCI secondary to cerebral vasospasm emerges 4 to 14 days following aSAH, leading to a decline in mean cerebral blood flow and further exacerbating microcirculatory dysfunction and white matter tract injury (Maher et al., 2020; Thilak et al., 2024). During this distinct DCI window, the initially altered microbiome ceases to be a mere downstream consequence and transitions into an active potential modulator. The continuous influx of shifted microbial metabolites (such as depleted SCFAs and accumulated BCAAs) is hypothesized to act as a sustained “secondary hit,” actively impairing neural repair, potentially exacerbating delayed ischemic damage, and laying the pathological foundation for long-term cognitive impairment. Radiologically, manifestations of aSAH can lead to diffuse brain injury, characterized by global reductions in gray and white matter volumes. It can also cause focal lesions that strongly correlate with specific cognitive deficits, particularly executive dysfunction (Al-Khindi et al., 2010).

However, aSAH presents not merely as a localized central nervous system catastrophe but also triggers a profound systemic stress and inflammatory response (Al-Mufti et al., 2019). This intense systemic inflammatory storm disrupts the pre-existing immune homeostasis of the host, serving as a potential modulatory bridge that links the central injury to intestinal dysfunction. Recently, with the continuous deepening of research on the intestinal microecology, the role of the intestinal microbiota in neurological disorders has received increasing attention. Accumulating evidence indicates that intestinal microbiota imbalance is inextricably linked to an array of cognitive-related conditions, including schizophrenia spectrum disorders, Alzheimer’s disease, and vascular dementia, with specific microbiota serving as potential cognitive biomarkers (Frileux et al., 2024; Liu et al., 2025; Warren et al., 2026). Therefore, it is crucial to explore the mechanism of the role of the intestinal microbiota in cognitive impairment after aSAH.

## 3 Characteristic changes in gut microbiota following aSAH

The human intestinal tract harbors a highly diverse microbial ecosystem, comprising hundreds of distinct species that collectively generate a large number of metabolites. Among these microorganisms, the phyla Bacteroidetes and Firmicutes are overwhelmingly dominant, accounting for over 90% of the total gut microbial population (Qin et al., 2010). Fan and Pedersen (2020) found that high diversity and genetic richness are quintessential hallmarks of a healthy gut microbiome. Conversely, the richness and diversity of the intestinal microbiota in patients with cognitive impairment have decreased (Xi et al., 2021).

The various physiological changes that occur rapidly in patients after aSAH are highly correlated with the rapid onset of gut microbial dysbiosis. During the acute phase, the systemic stress response robustly activates the sympathetic nervous system and the hypothalamic–pituitary–adrenal (HPA) axis. This neuroendocrine activation profoundly affects gastrointestinal motility and secretory functions, subsequently shifting the microbiome composition (Singh et al., 2016; Morais et al., 2020). Notably, current clinical cohort studies investigating the gut microbiota following aSAH predominantly focus on the acute phase, during which patients typically undergo systemic treatment in the neuro-intensive care unit (NICU). Within this specific clinical environment, iatrogenic factors such as the surgical trauma itself, the prophylactic administration of broad-spectrum antibiotics, and enteral nutrition interventions serve as significant confounding variables that drive alterations in the microbial community (Singh et al., 2016). Moving forward, rigorous longitudinal cohort studies are urgently warranted to eliminate these confounding factors and precisely identify disease-specific microbial signatures.

Recent investigations have revealed significant alterations in the *β*-diversity of the gut microbiota between aSAH patients and those with unruptured intracranial aneurysms (UIAs) (unweighted UniFrac: *p* = 0.013). Notably, the abundance of the genus *Campylobacter*, specifically *Campylobacter ureolyticus*, was found to be significantly elevated in the aSAH cohort compared to patients with UIAs (Kawabata et al., 2022). Xu et al. (2024) further pointed out that the *α*-diversity of the intestinal microbiota in aSAH patients decreased, with a depletion in the phylum Firmicutes (*p* < 0.05) and an expansion in the phylum Proteobacteria (*p* < 0.05), and it was found that the abundances of beneficial bacteria, including *Faecalibacterium*, *Roseburia*, *Metaproteus*, and *Blautia*, were significantly lower in aSAH patients than in the unruptured group (*p* < 0.05). Csecsei et al. (2025) also reported profound differences in β-diversity between aSAH and UIA patients (Bray-Curtis: *p* = 0.020; unweighted UniFrac: *p* = 0.029). Their analysis indicated that aSAH patients exhibited an enrichment of putatively pro-inflammatory bacteria, such as *Anaerotruncus*, *Faecalibacterium*, *Sellimonas*, and *Hungatella*. Conversely, patients with UIAs harbored a higher abundance of butyrate-producing bacteria, including *Faecalibacterium prausnitzii*, *Roseburia*, *Agathobacter*, and members of the Clostridiaceae. Collectively, they may maintain the stability of the aneurysm through the anti-inflammatory effect of butyric acid. Additionally, Sun et al. (2024) observed a significant enrichment of *Klebsiella pneumoniae*, *Ruminococcus gnavus*, and several well-known indole-producing bacteria (e.g., Enterobacteriaceae and *Alistipes*) in aSAH patients. In contrast, the abundance of beneficial microbiota, specifically *Bifidobacterium breve* and *Bifidobacterium pseudocatenulatum*, was significantly decreased.

In summary, after aSAH, the intestinal microbiota shows changes such as decreased diversity, enrichment of pro-inflammatory bacteria, and reduction of beneficial bacteria, particularly butyrate-producing bacteria (Table 1). Such shifts in the microbial community structure present not merely as an accompanying phenomenon of the disease but bear profound pathological significance. For instance, the severe depletion of butyrate-producing microbiota directly attenuates the neuroprotective and anti-inflammatory effects of gut-derived short-chain fatty acids (SCFAs), a phenomenon widely demonstrated to correlate significantly with impaired executive function and spatial memory decline across various models of cognitive impairment (Koh et al., 2016; Xu et al., 2026; Zheng et al., 2026). Furthermore, the enrichment of pro-inflammatory pathogenic bacteria is associated with an elevated circulating endotoxin burden, which may provide a pathological foundation for the subsequent development of cognitive dysfunction.

**Table 1:** Characteristic alterations of gut microbiota in aSAH.

Gut microbiota taxa	Alteration	Potential pathological role	References
Firmicutes and Firmicutes/Proteobacteria ratio	Decrease	Reflects macroscopic intestinal flora imbalance and weakens the anti-inflammatory effect of the microbial community; highly correlated with metabolic disorders of unsaturated fatty acids (e.g., *α*-linolenic acid) and essential amino acids (e.g., proline), potentially leading to decreased vascular elasticity.	Xu et al. (2024)
Campylobacteriota, particularly *Campylobacter* (specifically *Campylobacter ureolyticus*)	Increase	Induces systemic inflammatory responses; increases the release of IL-8, MMP-8, MMP-9, and neutrophil elastase, promoting vascular remodeling and cell death in the cerebral aneurysm wall.	Kawabata et al. (2022)
SCFA-producing bacteria (*Faecalibacterium*, *Roseburia*, *Blautia*, *Lachnospira*, *Agathobaculum*)	Decrease	Significantly reduces anti-inflammatory metabolites (e.g., butyrate, acetate); impairs intestinal barrier integrity, reduces inhibition of the NF-κB inflammatory pathway, and weakens vascular endothelial protection.	Xu et al. (2024) and Csecsei et al. (2025)
Tryptophanase/Indole-producing bacteria (*Klebsiella pneumoniae*, Enterobacteriaceae, *Alistipes*, *Ruminococcus gnavus*)	Increase	Leads to abnormal activation of tryptophan metabolism, significantly elevating the harmful metabolite IS; causes vascular endothelial dysfunction, upregulates MMP-9 expression, and accelerates elastin degradation in cerebral arteries.	Sun et al. (2024)
Potentially pathogenic and pro-inflammatory bacteria (Proteobacteria, *Anaerotruncus*, *Coprobacillus*, *Hungatella*, *Sellimonas*)	Increase	Associated with poor cardiovascular and neurological outcomes; promotes inflammation via the peptidoglycan biosynthesis pathway, exacerbating inflammatory responses and pathological remodeling of the vascular wall.	Csecsei et al. (2025) and Xu et al. (2024)
Specific Probiotics (*Bifidobacterium breve*, *Bifidobacterium pseudocatenulatum*)	Decrease	Drastically reduces the abundance of specific core probiotics, potentially impairing intestinal homeostasis and host systemic immune regulation.	Sun et al. (2024)

Crucially, previous observational studies have failed to establish a definitive causal relationship between baseline gut dysbiosis and the initial rupture of intracranial aneurysms. To delineate the long-term cognitive impairment following aSAH, it is imperative to explicitly define the dynamic role of the microbiome: whether it acts as a primary cause, a downstream consequence, or an active modulator. Based on current cross-sectional clinical evidence, the acute shift in gut microbiota is primarily a consequence of the profound systemic stress and early brain injury induced by the hemorrhagic event. However, we postulate that this secondary dysbiosis does not remain a passive bystander. Instead, it acts as a potential modulator. Through the release of altered metabolites, it may impose a “secondary hit” on the brain tissue. This secondary hit is hypothesized to aggravate DCI and is implicated in the long-term trajectory of cognitive decline. Therefore, while strict causality in humans remains to be fully elucidated, the microbiome serves as a compelling modulatory target requiring further validation.

## 4 Potential pathways of gut microbiota and their metabolites in post-aSAH cognitive impairment

### 4.1 Immune-inflammatory pathway

The intestinal mucosal immune system, through continuous exposure to a large number of microbial molecules, has evolved a highly regulated dynamic equilibrium. It can not only maintain immune tolerance to commensal microorganisms but also effectively defend against pathogens, protecting the integrity of the host. Under the regulation of the gut microbiota, a delicate balance between regulatory T cells (Tregs) and effector T cells (Teffs) is meticulously maintained (Ivanov and Littman, 2011). However, aSAH-induced intestinal dysbiosis disrupts this homeostasis, triggering the systemic activation of peripheral effector T cells. The inflamed central nervous system (CNS) subsequently recruits these sensitized lymphocytes. Early post-aSAH, the intracranial microvasculature significantly upregulates C-X-C motif chemokine ligand 12 (CXCL12), utilizing the CXCL12/C-X-C chemokine receptor 4 (CXCR4) axis to guide hyperactive peripheral CD8 + T cells across the blood–brain barrier (BBB) (Li et al., 2025). Clinical phenotyping of human SAH aneurysm walls corroborates this, showing a dense infiltration of cytotoxic, polyfunctional T cells secreting copious interferon-*γ* (IFN-γ), tumor necrosis factor-*α* (TNF-α), and interleukin-2 (IL-2) (Moschetti et al., 2022). Upon infiltration, these T cells release perforin and granzyme B to induce endothelial apoptosis and exacerbate BBB breakdown (Li et al., 2025). This irrevocably amplifies the neuroinflammatory cascade, a pathological event strongly linked to the progression of cognitive impairment, although its exact causal contribution requires further validation.

As the principal resident immune cells of the CNS, microglia exert a highly dynamic and dichotomous role in neuroinflammation following aSAH. Specifically, they transition between pro-inflammatory (M1-like) states that initiate the inflammatory cascade and anti-inflammatory (M2-like) phenotypes that facilitate hematoma clearance and tissue repair (Zheng et al., 2020). Erny et al. (2017) found that microglia exhibit an immature phenotype in germ-free mice, revealing the gut microbiota as an indispensable driver of microglial homeostasis and highlighting microbiota-targeted interventions as a viable strategy to modulate microglial activity. Following aSAH, elevated levels of branched-chain amino acids (BCAAs), particularly leucine, are speculated to drive microglia to enter a transitional phenotype distinct from classical M1 or M2 polarization states. While characterized by enhanced phagocytic capacity, this intermediate state presents with increased levels of the oxidative stress marker 15-F2t-isoprostane (15-F2t-IsoP) and a concomitant decline in neuroprotective mediators such as insulin-like growth factor-1 (IGF-1). Upon stimulation with LPS, these cells mount a strong secretory response, releasing large amounts of pro-inflammatory cytokines, including TNF-*α*, interleukin-6 (IL-6), and interleukin-1β (IL-1β) (De Simone et al., 2013; Savarraj et al., 2022).

Distinct from ischemic stroke, the pathological hallmark of aSAH lies in the direct neurotoxicity exerted by subarachnoid blood accumulation and its degradation products, including heme and hemosiderin (Loftspring, 2010). Under the stress condition of aSAH, the increased permeability of the intestinal barrier may allow Gram-negative bacteria and their cell wall component, LPS, to enter the systemic circulation, thereby triggering both systemic and neuroinflammation (Bello-Corral et al., 2023). Due to the early damage of the BBB in aSAH, circulating LPS can readily penetrate the cerebral parenchyma. Notably, heme released from the subarachnoid hemorrhage serves as an endogenous ligand for Toll-like receptor 4 (TLR4). The concurrent presence of gut-derived exogenous LPS and brain-accumulated endogenous heme is hypothesized to exert a profound synergistic activation of TLR4 on microglia, inducing the explosive secretion of pro-inflammatory factors such as TNF-*α*, IL-1β, and IL-6 by microglial cells (Lu et al., 2018; Liu G. J. et al., 2020).

**Figure 1:**
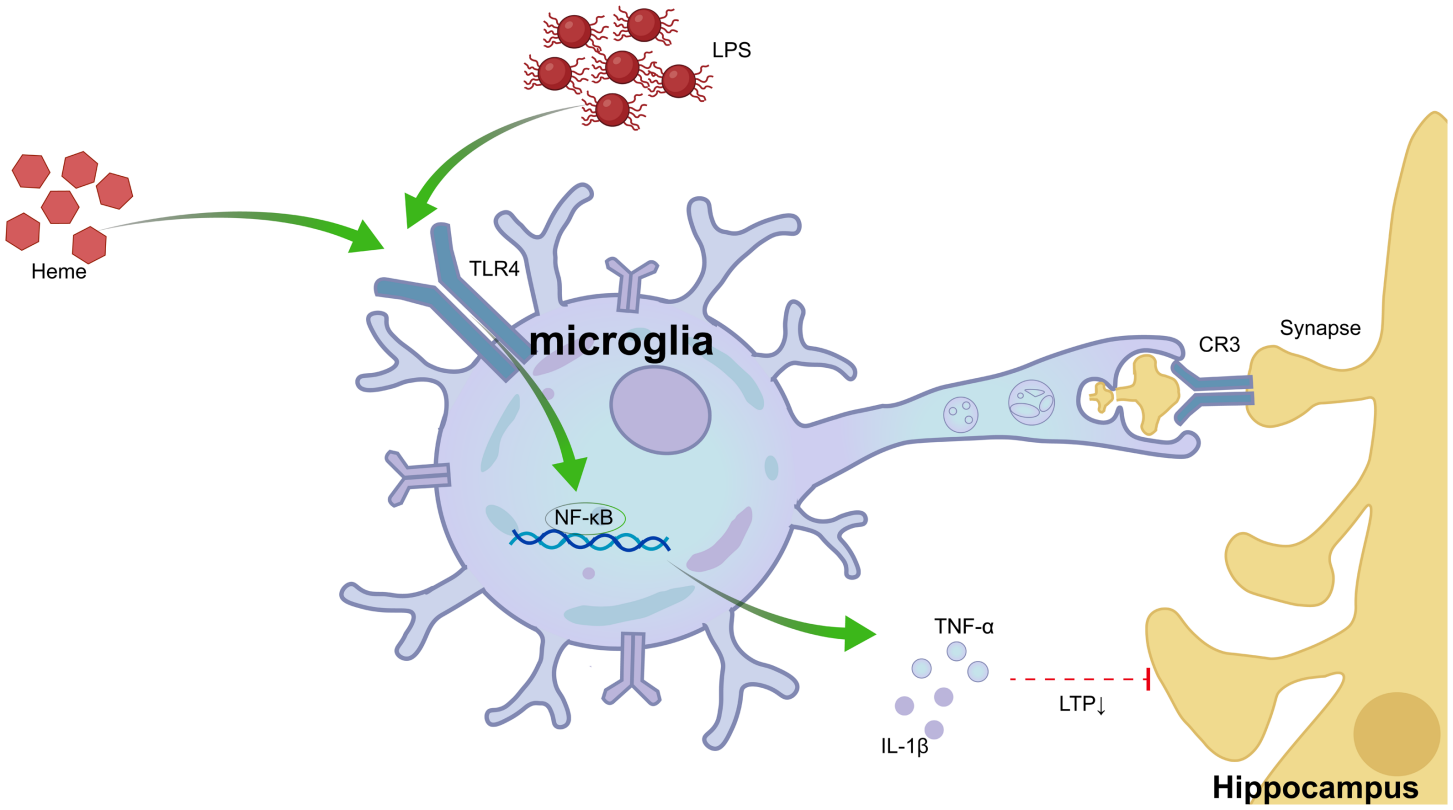
Gut dysbiosis-driven neuroinflammation and synaptic dysfunction post-aSAH. Circulating LPS and endogenous heme synergistically activate microglial TLR4, triggering the release of pro-inflammatory cytokines. This hyperactivation is associated with microglial CR3 overexpression, which may mediate excessive excitatory synapse pruning. Furthermore, elevated inflammatory cytokines block hippocampal LTP.

Such hyperactivated microglia not only propagate neuroinflammation but also have the potential to interfere with neural circuit remodeling. Under physiological conditions, microglia are actively involved in delicate synaptic pruning. Importantly, clinical evidence shows early complement proteins, specifically complement component 1q (C1q) and complement component 3 (C3), are significantly elevated in the cerebrospinal fluid of aSAH patients, correlating with neurological outcomes (Geraghty et al., 2026). Following aSAH, gut-derived exogenous endotoxins (e.g., LPS) and brain-accumulated endogenous heme act as a potent dual-inflammatory trigger that synergistically upregulates microglial complement receptor 3 (CR3). Driven by the accumulated C1q and C3 ligands, this overactivated CR3 pathway accelerates aberrant phagocytosis and excitatory synapse loss in the hippocampus. Furthermore, elevated concentrations of TNF-α and IL-1β have been demonstrated to directly block the induction of long-term potentiation (LTP) in the hippocampal CA1 region—the fundamental electrophysiological mechanism underlying learning and memory formation (Yang et al., 2022; Du et al., 2023). Consequently, neuroinflammation associated with gut microbiota dysbiosis may be associated with memory encoding deficits and executive function decline in aSAH patients by potentially disrupting hippocampal synaptic structure and functionally inhibiting synaptic plasticity (Figure 1).

Patients with aSAH exhibit increased abundance of tryptophanase genes and indole-producing bacteria, notably *Klebsiella pneumoniae* and *Ruminococcus*, along with significantly elevated plasma levels of tryptophan (TRP)-derived metabolites, including indoxyl sulfate (IS), kynurenine (KYN), and serotonin (5-HT) (Sun et al., 2024). These TRP metabolites mainly participate in immune regulation by activating the signaling pathway through the transcription factor aryl hydrocarbon receptor (AhR) signaling, promoting the differentiation of anti-inflammatory macrophages, Tregs, and regulatory B cells that secrete interleukin-10 (IL-10) and interleukin-35 (IL-35), playing a crucial role in the maintenance of both intestinal and systemic homeostasis (Zádori et al., 2011; Lee et al., 2016; Gargaro et al., 2019; Su et al., 2022).

Furthermore, the dysbiosis observed post-aSAH is characterized by a concurrent depletion of SCFAs (Xu et al., 2024; Csecsei et al., 2025), which significantly compromises their native anti-inflammatory capacities. Studies have found that elevated levels of acetate and butyrate are associated with decreased phosphorylation levels of NF-κB p65 and IκB, thereby exerting anti-inflammatory effects (Sun et al., 2019; Liu J. et al., 2020). Moreover, acetate can improve the cognitive function of patients by regulating signaling pathways such as interleukin-18 (IL-18) (Nguyen et al., 2024).

Furthermore, the post-aSAH depletion of BAs diminishes their efficacy in stimulating the release of glucagon-like peptide-1 (GLP-1). GLP-1 can cross the blood–brain barrier into the CNS, bind to corresponding receptors, and exert neuroprotective and anti-inflammatory responses (Mertens et al., 2017; Li et al., 2021; Sun et al., 2023). Simultaneously, specific BAs such as tauroursodeoxycholic acid (TUDCA) activate the GPBAR1 (also known as TGR5) receptor, thereby regulating the cyclic adenosine monophosphate (cAMP)/protein kinase A (PKA) signaling pathway to inhibit the pro-inflammatory NF-κB pathway and the NOD-like receptor pyrin domain-containing 3 (NLRP3) inflammasome, while driving the transformation of microglia to an anti-inflammatory phenotype (Guo et al., 2016; Yanguas-Casás et al., 2017). Thus, the marked decline in BA levels is hypothesized to substantially impair these critical neuroinflammatory regulatory mechanisms within the specific pathological context of aSAH.

In summary, the dysbiosis of the microbiota and the changes in its metabolites after aSAH are strongly associated with exacerbated cerebral injury and are implicated as modulators in the progression of cognitive impairment by compromising intestinal barrier integrity, disrupting microglial homeostasis, and modulating multifaceted inflammatory signaling pathways.

### 4.2 Neuroendocrine and neurotransmitter pathways

A bidirectional interplay exists between the gut microbiota and the neuroendocrine signaling pathways mediated by the HPA axis. Specifically, the stress response triggered during the acute phase of aSAH activates the HPA axis, which impacts gastrointestinal function and subsequently alters the composition of the gut microbiota. Conversely, the depletion of gut microbiota disrupts HPA axis function, leading to a reduction in both neurotrophic factors and neurotransmitters (Prenderville et al., 2015; Morais et al., 2020).

The gut microbiota can directly synthesize or modulate the levels of neurotransmitters, such as *γ*-aminobutyric acid (GABA) and 5-HT. Microbiota-derived metabolites, including SCFAs and indole, act on enteroendocrine cells to regulate the secretion of neuropeptides like GLP-1, and influence the release of neuromodulators that possess dual hormonal and neurotransmitter functions, such as 5-HT, which ultimately affects emotional and behavioral responses (Dinan and Cryan, 2017; Gonzalez-Arancibia et al., 2019; Morais et al., 2020).

Although an appropriate amount of BCAAs is beneficial for neurotransmitter synthesis and energy metabolism under normal physiological conditions, we observed that abnormal elevations in plasma BCAA concentrations are frequently associated with poor prognosis in the pathological state of aSAH (Savarraj et al., 2022). While this phenomenon was previously attributed primarily to the host’s hypercatabolic state, studies have shown that the gut microbiota may serve as a crucial source of circulating BCAAs (Newgard et al., 2009; White and Newgard, 2019). Notably, BCAAs can enter the brain via the large neutral amino acid transporter 1 (LAT1) located on the blood–brain barrier. Given that other large neutral amino acids, such as TRP and tyrosine (TYR), share this LAT1 transport mechanism, chronic and excessive BCAA accumulation may competitively inhibit the translocation of TRP and TYR across the blood–brain barrier. This competitive inhibition is speculated to subsequently deplete central 5-HT and dopamine (DA) levels (Le Masurier et al., 2005; Coppola et al., 2013; Polis and Samson, 2020).

Both neurotransmitters are integral to regulating the expression of brain neurotrophic factors. An imbalance in these transmitters can trigger a systemic disruption of neurotrophic support, which is correlated with the progression of neurodegenerative diseases and the development of cognitive impairment (Le Masurier et al., 2005; Coppola et al., 2013; Prenderville et al., 2015). The suppression of DA synthesis, in particular, may be intimately linked to the executive dysfunction and anhedonia commonly observed in aSAH patients, which requires further validation. Additionally, DA has been shown to improve cognitive impairment (Bonet Olivares et al., 2025). Concurrently, the restricted intake of TRP is associated with a decrease in 5-HT levels within the hippocampus and limbic system. This depletion not only precipitates anxiety and depression but is also hypothesized to directly attenuate 5-HT-mediated synaptic plasticity and neurogenesis, ultimately contributing to emotional memory deficits and the loss of adaptive behaviors (Han et al., 2018).

### 4.3 Neural pathways

The gut microbiota can influence the central nervous system via the vagus nerve, under both physiological and pathological conditions, being hypothesized as capable of transmitting intestinal microbial signals to the brain and also reversing the signals from the brain to the gut (Spielman et al., 2018).

The vagus nerve represents the most important neural pathway for bidirectional communication between the gut microbiota and the brain. Bravo et al. (2011) first demonstrated in a healthy mouse model that the chronic ingestion of *Lactobacillus* can regulate the expression of GABA receptor subtypes (GABAB1b and GABAα2) in critical brain regions—including the prefrontal cortex, hippocampus, and amygdala—through the vagus nerve, thereby effectively alleviating anxiety and depression-like behaviors.

The vagus nerve serves as a critical immune brake via the cholinergic anti-inflammatory pathway (CAP). It is widely hypothesized that its efferent fibers release acetylcholine, which binds to α7 nicotinic acetylcholine receptors (α7nAChR) on the surface of macrophages, thereby inhibiting the release of pro-inflammatory cytokines (Pereira and Leite, 2016; Ye et al., 2026). In the pathological state of aSAH, the acute elevation of intracranial pressure coupled with the damage to the hypothalamus precipitates sympathetic overactivation. Concurrently, the parasympathetic nervous system undergoes profound suppression. This sustained inhibition of vagal tone leads to the failure of the CAP pathway, ultimately predisposing the host to a persistent inflammatory state. Encouragingly, counteracting this suppression holds therapeutic promise, as transcutaneous auricular vagus nerve stimulation (taVNS) safely restores autonomic balance in aSAH patients, and targeted α7nAChR activation attenuates early brain injury in aSAH models (Duris et al., 2011; Tan et al., 2025). Moreover, emerging evidence indicates that under stress conditions, dysbiosis of the gut microbiota can trigger changes in neurohormones via the vagus nerve, sustaining inflammatory responses and contributing to cellular apoptosis, providing a hypothesized pathological foundation for cognitive impairment and dementia (Ticinesi et al., 2018).

### 4.4 Oxidative stress

Gut dysbiosis serves as a potential source of oxidative stress, and the exacerbation of oxidative stress may further contribute to the progression of cognitive impairment (Prenderville et al., 2015; Bello-Corral et al., 2023). Following aSAH, the breakdown of extravasated blood releases substantial amounts of heme and ferrous iron (Fe^2+^), triggering an explosive oxidative response. Iron ions can catalyze lipid peroxidation via the Fenton reaction, ultimately precipitating ferroptosis—an iron-dependent form of programmed cell death (Hirschhorn and Stockwell, 2019). It is imperative to recognize how these aSAH-specific pathological events may interact synergistically with microbiome-derived metabolite shifts to drive cognitive decline. Glutathione peroxidase 4 (GPX4) is the core enzyme in the cellular defense against ferroptosis (Dixon and Stockwell, 2019). In patients with aSAH, microbial dysbiosis leads to a marked reduction in SCFAs, particularly butyrate (Xu et al., 2024; Csecsei et al., 2025). As a highly potent inhibitor of histone deacetylases (HDACs), butyrate activates the nuclear factor erythroid 2-related factor 2 (Nrf2) antioxidant pathway (Haase et al., 2018). Under physiological conditions, Nrf2 initiates the transcription of GPX4 and superoxide dismutase 1 (SOD1) to suppress ferroptosis and clear lipid peroxides (Kim et al., 2020; Wang C. et al., 2022). It is hypothesized that post-aSAH depletion of gut-derived butyrate compromises the neuronal defense against this “hemorrhagic oxidative insult,” potentially resulting in extensive hippocampal neuronal death and subsequent cognitive function decline. Specifically, precisely when the brain is inundated with iron overload and subsequent Fenton reactions triggered by subarachnoid blood, the microbiome-driven butyrate depletion is hypothesized to critically compromise the GPX4/Nrf2 antioxidant defenses. This proposed lethal synergy between aSAH-induced oxidative insults and the microbiome-driven loss of antioxidant defenses may render neurons highly susceptible to lipid peroxidation and irreversible ferroptosis.

Studies have shown that plasma TRP metabolites are significantly elevated in patients with aSAH (Sun et al., 2024). High doses of TRP can induce oxidative stress, which may lead to the accumulation of neurotoxic metabolites and the exacerbation of related symptoms (Hiratsuka et al., 2013). Under the stress state of aSAH, the kynurenine metabolic pathway is triggered, correlating with the increased production of the neurotoxic metabolite quinolinic acid (QA) (Trebaticka et al., 2025). As a potent N-methyl-D-aspartate (NMDA) receptor agonist, QA drives excitotoxicity by promoting Ca^2+^ influx and directly induces the generation of reactive oxygen species (ROS) (Lugo-Huitrón et al., 2013). The initial hemorrhagic insult of aSAH is inherently accompanied by pathologically elevated extracellular glutamate (GLU) levels in the cerebrospinal fluid. The synergistic effect between gut-derived QA and this excessive endogenous GLU is speculated to render NMDA receptor-rich hippocampal neurons extremely susceptible to excitotoxic and lethal injury, though this requires further *in vivo* validation (Zhang et al., 2018).

**Figure 2:**
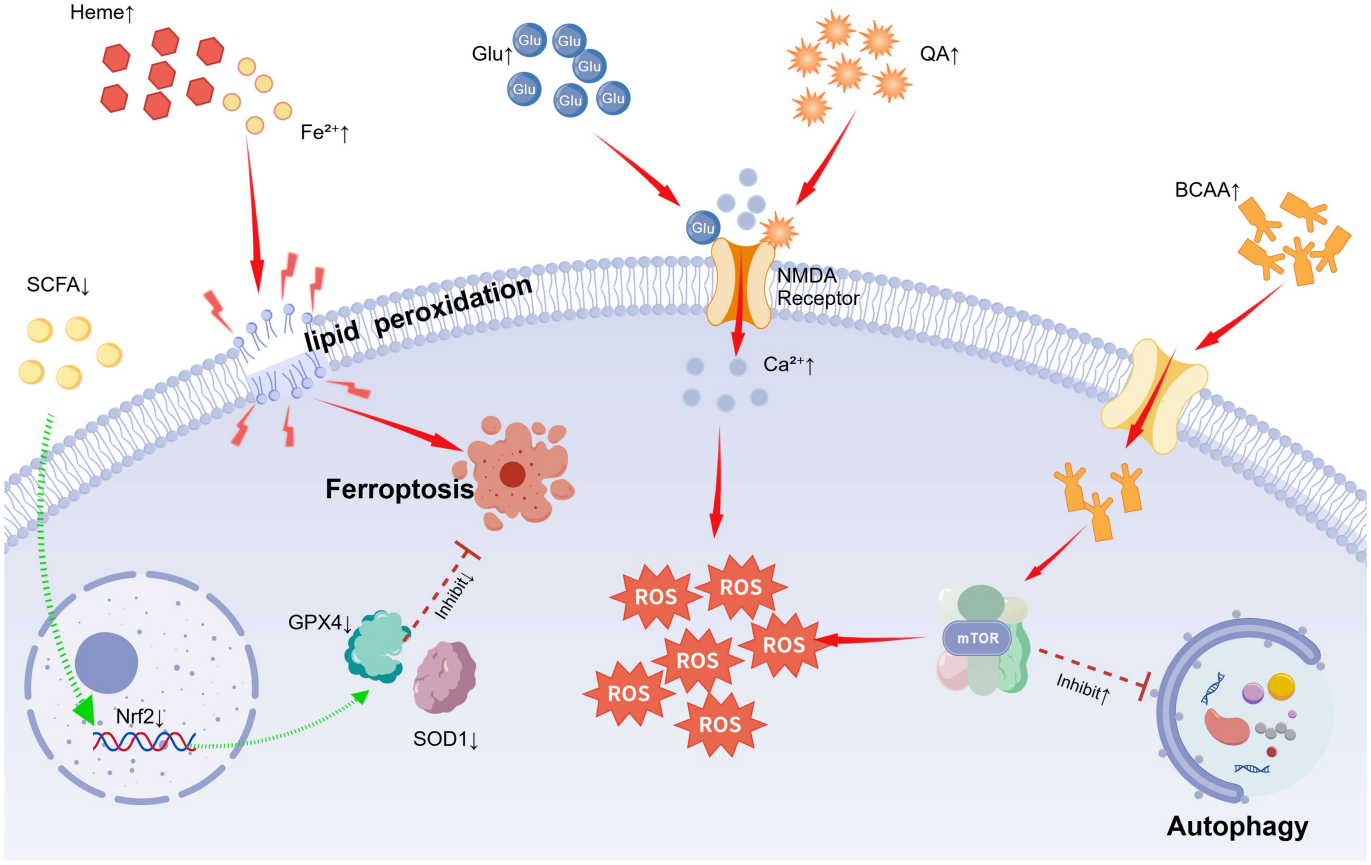
Microbiota metabolites modulate oxidative stress and autophagy post-aSAH. Subarachnoid blood degradation releases iron, driving lipid peroxidation and ferroptosis. The depletion of gut-derived butyrate impairs the Nrf2/GPX4 antioxidant defense. Concurrently, elevated QA and glutamate synergistically exacerbate NMDA receptor excitotoxicity and ROS production. Furthermore, excessive BCAAs activate mTOR, which correlates with the suppression of neuronal autophagy. These metabolic shifts collectively modulate and potentially prolong oxidative stress. Created with BioGDP.com (Jiang et al., 2025).

In addition to stimulating muscle protein synthesis via the mammalian target of rapamycin (mTOR) pathway, elevated BCAAs following aSAH are postulated to directly induce neuronal excitotoxicity through mTOR activation (Carunchio et al., 2010; Savarraj et al., 2022). Zhenyukh et al. (2017) demonstrated that high BCAA concentrations can also activate the phosphoinositide 3-kinase (PI3K)/ protein kinase B (Akt)/ mammalian target of rapamycin complex 1 (mTORC1) pathway, increasing the generation of nicotinamide adenine dinucleotide phosphate hydrogen oxidase 1 (NOX-1), nicotinamide adenine dinucleotide phosphate hydrogen oxidase 2 (NOX-2) and mitochondrial ROS. Notably, even at lower concentrations, BCAAs can exacerbate oxidative stress when present in inflammatory or hyperglycaemic conditions. mTOR is a principal negative regulator of autophagy (Caza et al., 2022). Following aSAH, neurons typically rely on autophagic mechanisms to clear damaged mitochondria and neurotoxic hemoglobin degradation products. However, the sustained activation of mTOR driven by gut-derived BCAAs suppresses this autophagic response, potentially leading to the toxic accumulation of these specific hemorrhagic byproducts and oxidized proteins, thereby hypothetically exacerbating oxidative stress. Beyond the direct generation of ROS, this autophagic inhibition is hypothesized to prolong oxidative stress from the acute into the chronic phase, ultimately contributing to delayed neurodegenerative alterations and cognitive impairment (Figure 2). This is theorized to create a lethal synergistic environment: the gut-derived BCAA accumulation sustains the toxic burden of aSAH-specific hemoglobin products, while the concurrent butyrate depletion removes the vital antioxidant shields.

### 4.5 Apoptosis

Following aSAH, alterations in the cerebral microenvironment and microcirculatory disorders can cause damage to subcellular organelles, such as mitochondria and the endoplasmic reticulum, thereby potentially culminating in cellular apoptosis (Fang et al., 2018). Furthermore, gut microbiota dysbiosis is hypothesized to precipitate neuroinflammation and oxidative stress, thereby creating a microenvironment conducive to neuronal injury and accelerated apoptosis. Extensive neuronal apoptosis is a well-established correlate of irreversible cognitive impairment (Mu et al., 2022). Notably, aSAH is additionally characterized by pyroptosis, a severe form of inflammatory cell death.

Following the disruption of the intestinal barrier, gut-derived LPS translocates into the brain, acting as a pathogen-associated molecular pattern (PAMP), and is a classic activator of the NLRP3 inflammasome (Kelley et al., 2019). Upon binding to its receptors, LPS promotes the assembly of the NLRP3 inflammasome and the subsequent activation of caspase-1, which subsequently cleaves Gasdermin D (GSDMD) protein to form a membrane pore, causing the cell membrane to rupture and releasing IL-1β and IL-18 (Kelley et al., 2019). This pyroptotic cascade driven by gut-derived endotoxins is postulated to not only directly induce neuronal lysis but also amplify local neuroinflammation, thereby establishing a vicious cycle that awaits further clinical validation in aSAH patient cohorts.

TUDCA, as a secondary bile acid strictly dependent on the metabolic activity of the gut microbiota, is a natural neuroprotective agent bridging the gut-brain axis (Palmela et al., 2015). Crucially, unlike mechanisms extrapolated from other neurological disorders, the neuroprotective efficacy of TUDCA has been directly validated within specific experimental models of aSAH (Wu et al., 2020). In experimental models of aSAH, TUDCA has been demonstrated to penetrate the blood–brain barrier and specifically activate the membrane receptor TGR5 (GPBAR1).

This activation not only robustly inhibits the assembly of the NLRP3 inflammasome, thereby abrogating pyroptosis, but also stabilizes mitochondrial membranes via the cAMP-PKA-CREB signaling pathway. Furthermore, TUDCA regulates the expression of downstream apoptosis-associated proteins, such as B-cell lymphoma 2 (BCL-2), BCL-2-associated X protein (BAX), and caspase-3, thereby significantly attenuating hippocampal neuronal apoptosis and conferring substantial neuroprotection (Guo et al., 2016; Wu et al., 2020). Guo et al. (2023) discovered that butyrate modulates the PI3K/AKT/caspase-3 signaling pathway to suppress neuronal apoptosis, and it is hypothesized that the reduction of butyrate after aSAH weakens this neuroprotective effect.

### 4.6 BBB disruption

Germanó et al. (1992) first used [^14^C]-*α*-aminoisobutyric acid technology to prove that the blood–brain barrier permeability after SAH significantly increased, especially in the cerebral cortex and cerebellar gray matter regions, with the permeability being 1.3 to 1.5 times that of the control group. During the EBI phase of aSAH, the explosive expression of matrix metalloproteinase 9 (MMP9) represents a crucial molecular event precipitating BBB disruption (Hosokawa et al., 2011; Triglia et al., 2016). Following the impairment of the gut barrier, LPS and its induced inflammatory factors entering the systemic circulation can further compromise BBB integrity. Upon reaching the cerebral vascular endothelium via the circulation, LPS can further upregulate the expression and activity of MMP9 in vascular endothelial cells and pericytes via the TLR4/NF-κB pathway (Aid et al., 2010; Megur et al., 2020). The highly active MMP9 specifically degrades tight junction proteins constituting the BBB, particularly Claudin-5, potentially resulting in the abnormal opening of endothelial intercellular spaces (Chen et al., 2024).

The metabolic products of intestinal symbiotic bacteria should be an important guarantee for maintaining the integrity of the BBB. Propionate, acting through its receptor free fatty acid receptor 3 (FFAR3) to activate Nrf2 signaling, effectively shields the BBB from oxidative stress-induced damage (Hoyles et al., 2018). Furthermore, it synergizes with other SCFAs to modulate tight junction proteins, thereby preserving BBB integrity (Logsdon et al., 2017). However, the significant dysbiosis of gut microbiota in patients with aSAH is associated with a diminished abundance of SCFA-producing bacteria. Compromised cerebral vascular endothelial cells become increasingly vulnerable to MMP9-mediated degradation and oxidative damage, ultimately resulting in delayed repair or even permanent disruption of the BBB, a concept that requires further clinical validation.

In neuroinflammatory diseases, alterations in BBB permeability facilitate the translocation of microbial products and drive the infiltration of peripheral immune cells into the brain parenchyma. Conversely, brain inflammation caused by diseases such as stroke alters the composition of the gut microbiome, establishing a vicious cycle where dysbiosis acts as a potential modulator that speculatively amplifies neuroimmune responses. The loss of BBB integrity allows gut-derived neurotoxins, including high concentrations of LPS, indoxyl sulfate (IS), and pro-inflammatory cytokines, to directly infiltrate the hippocampal parenchyma and prefrontal cortex, ultimately hypothesized to aggravate pathological damage and cognitive impairment (Benakis et al., 2020; Xu et al., 2023).

In summary, the dysbiosis and altered metabolic profiles following aSAH disrupt the homeostatic balance between the gut and the central nervous system. These shifts are postulated to contribute to neurological deficits and cognitive decline through multiple pathways, including the promotion of neuroinflammation, exacerbation of oxidative stress, potential induction of neuronal apoptosis, impairment of BBB integrity, and interference with neurotransmitter systems. A comprehensive understanding of these complex mechanisms not only provides a novel perspective on the pathophysiology of post-aSAH cognitive impairment but also establishes a critical theoretical foundation for developing future gut microbiota-targeted therapeutic strategies.

To clarify the current state of knowledge and prevent the conflation of evidence, we have stratified the multifaceted mechanistic statements discussed above into a dedicated evidence grading table (Table 2). This explicit stratification categorizes the biological mechanisms into three distinct tiers: direct clinical evidence derived from aSAH patients, preclinical evidence specific to aSAH animal models, and mechanisms extrapolated from other neurological diseases or general physiological principles. By delineating what has been empirically validated within the unique aSAH microenvironment from what remains a theoretical extrapolation, we aim to highlight critical knowledge gaps.

**Table 2:** Comprehensive stratification of mechanistic evidence in post-aSAH cognitive impairment.

Evidence tier	Definition	Potential pathways	Comprehensive mechanistic statements
Tier 1: Direct evidence from aSAH patients	Mechanisms and biomarker shifts directly observed in human aSAH clinical cohorts.	Immune-inflammatory:	Dense infiltration of cytotoxic T cells secreting IFN-γ, TNF-α, and IL-2 in human SAH aneurysm walls. Elevated complement proteins (C1q and C3) in the CSF of aSAH patients.
		Metabolome shifts	Elevated plasma levels of TRP-derived metabolites (IS, KYN, 5-HT, QA) and increased indole-producing bacteria. Pathological elevation of plasma BCAAs correlating with poor prognosis. Significant depletion of gut-derived SCFAs (particularly butyrate). Depletion of circulating BAs.
		Neural	taVNS effectively increases parasympathetic activity and restores autonomic balance following aSAH.
Tier 2: Evidence from aSAH animal models	Mechanisms directly validated within aSAH-specific experimental *in vivo* models.	Immune-inflammatory	The CXCL12/CXCR4 axis guides hyperactive peripheral CD8 + T cells across the blood–brain barrier. Endogenous heme and exogenous LPS exert profound synergistic activation of TLR4 on microglia.
		Neural	α7nAChR activation attenuates early brain injury.
		Apoptosis	TUDCA penetrates the blood–brain barrier, specifically activates the TGR5 receptor, and inhibits the NLRP3 inflammasome to attenuate neuronal apoptosis.
		BBB disruption	Explosive expression of MMP9 precipitates BBB disruption during the EBI phase, with markedly increased BBB permeability.
Tier 3: Extrapolated evidence or speculative mechanisms	Mechanisms inferred from *in vitro* experiments or other disease models.	Immune-inflammatory:	Gut microbiota drives microglial homeostasis (germ-free mice). BCAAs drive microglia to an intermediate transitional state. TNF-α/IL-1β directly block hippocampal LTP induction. TRP metabolites activate the anti-inflammatory AhR signaling pathway. SCFAs inhibit the NF-κB inflammatory pathway. BAs stimulate neuroprotective GLP-1 release and activate the TGR5/cAMP/PKA pathway to inhibit the inflammasome, driving microglial anti-inflammatory polarization
		Neuroendocrine	BCAAs competitively inhibit TRP/TYR via LAT1, depleting central 5-HT and DA.
		Neural	Lactobacillus regulates GABA receptors via the vagus nerve.
		Oxidative stress	Butyrate activates Nrf2/GPX4 to suppress lipid peroxidation and ferroptosis. QA and endogenous GLU synergistically exacerbate NMDA receptor excitotoxicity. BCAAs activate mTOR, suppressing neuronal autophagy.
		Apoptosis	LPS activates the NLRP3 inflammasome, inducing pyroptosis. Butyrate modulates the PI3K/AKT/caspase-3 signaling pathway.
		BBB disruption	Propionate shields the BBB from oxidative stress via the FFAR3 receptor. LPS upregulates MMP9 expression via the TLR4/NF-κB pathway.

## 5 Therapeutic strategies targeting gut microbiota for aSAH-related cognitive impairment

Based on the complex association mechanism between the gut microbiota and cognitive impairment after aSAH, intervention strategies targeting the microbial community are crucial. However, to maximize translational feasibility, the current stage of evidence must be explicitly recognized. In terms of therapeutic strategies, probiotics, enteral nutrition support, and dietary modifications serve as direct modulatory approaches. Given the clinical reality that patients with severe aSAH frequently experience coma or dysphagia during the acute phase, traditional dietary interventions can be adapted into enteral nutrition formulations enriched with specific prebiotics or SCFAs (Thayabaranathan et al., 2022; Razzak et al., 2026). Research indicates that probiotic supplementation, including *Bifidobacterium*, can ameliorate cognitive deficits by regulating the abundance of intestinal microorganisms and regulating microbial metabolites, such as LPS, butyrate, and valerate (Yu et al., 2025). Crucially, the current evidence for probiotics and prebiotics in cognitive preservation is primarily restricted to preliminary human data in non-aSAH cognitive disorders and pre-clinical models. Additionally, dietary interventions designed to directly increase the intake of beneficial metabolites, notably SCFAs and BAs, can exert a positive impact on cognitive impairment in patients with aSAH. However, it should be explicitly recognized that the current evidence supporting this concept does not stem from dietary intervention models in aSAH, but rather from pre-clinical mouse models utilizing direct pharmacological administration (Chen et al., 2020; Liang et al., 2026). Fecal microbiota transplantation (FMT) represents another crucial therapeutic strategy. By transferring gut microbiota from healthy donors to patients with aSAH, FMT directly restores microbial homeostasis and normalizes the levels of beneficial metabolites, thereby enhancing synaptic plasticity (Cammarota et al., 2017). While aSAH-specific human clinical data for FMT remain absent, existing evidence predominantly stems from pre-clinical animal models. Li et al. (2020) found that oral gavage of live *Hungatella hathewayi* in aSAH mice elevated serum taurine levels, subsequently alleviating related vascular inflammation and tissue damage. These findings provide a compelling rationale for improving long-term neurological outcomes following DCI.

Pharmacological modulation provides a more targeted therapeutic approach. Supported exclusively by aSAH-specific experimental animal models, pharmacological studies have demonstrated that INT-777, a TGR5 receptor agonist, can mimic the neuroprotective effects of BAs by activating the TGR5/cAMP/PKA and cAMP/protein kinase C epsilon (PKCε)/aldehyde dehydrogenase 2 (ALDH2) signaling pathways (Zuo et al., 2019; Hu et al., 2021; Mu et al., 2022). This mechanism effectively inhibits NLRP3 inflammasome-mediated neuronal pyroptosis. Therefore, the early administration of TGR5 agonists may emerge as a crucial strategy to ameliorate early brain injury and preserve cognitive function. Notably, by attenuating the extensive loss of hippocampal neurons, this approach provides a vital structural foundation for the preservation of higher-order cognitive capabilities in patients.

Alternatively, neuromodulatory interventions, such as vagus nerve stimulation, can facilitate the improvement of cognitive function through the reverse regulation of the gut microbiota. Specifically, non-invasive taVNS can reactivate parasympathetic tone, promoting the release of acetylcholine and acting on macrophage α7nAChR, thereby downregulating the release of intestinal pro-inflammatory cytokines (Pereira and Leite, 2016; Elkholey et al., 2022). Building upon recent clinical evidence demonstrating that taVNS safely and effectively restores autonomic balance specifically in aSAH patients, its application as a targeted neuromodulatory therapy holds tremendous translational potential (Tan et al., 2025). The restoration of vagal activity via the CAP serves to block the systemic propagation of peripheral inflammation to the central nervous system, establishing a low-inflammatory microenvironment conducive to the repair of hippocampal neurons.

Notably, the specific clinical environment of the NICU must be fully considered when evaluating the feasibility of clinical translation. Although the targeted depletion of endotoxin-producing pathogenic bacteria via bacteriophages or narrow-spectrum antibiotics represents an ideal theoretical strategy, the clinical reality within the NICU frequently necessitates the prophylactic or therapeutic administration of broad-spectrum antibiotics in patients with aSAH due to the compounding risks of ventilator-associated pneumonia (VAP) or central nervous system infections (Wang R. et al., 2022; Pottie et al., 2024). The prevalence of such clinical prescribing practices presents a substantial barrier to the translation of narrow-spectrum antimicrobial strategies. Furthermore, given that patients in the acute phase of aSAH frequently present with compromised intestinal mucosal barriers and immunosuppression, the direct administration of probiotics or FMT carries a potential risk of inducing gut-derived bacteremia (Fang et al., 2025). Consequently, a clear distinction must be made between acute phase and chronic recovery strategies. During this highly vulnerable acute period, initial clinical management should prioritize the direct supplementation of downstream metabolites exhibiting neuroprotective properties, such as SCFAs or TUDCA. This “metabolite replacement therapy” emerges as a theoretically safer and potentially efficacious strategy that critically bypasses bacterial colonization hurdles and the risk of bacteremia. Conversely, during the chronic recovery phase of aSAH, the primary therapeutic focus shifts towards the reconstruction of the gut microbiota. After overcoming the vasospasm phase and achieving clinical stability, patients with residual cognitive impairment may benefit from probiotic preparations, prebiotic dietary interventions, or even FMT, as the aim is to safely reshape the diversity of healthy intestinal microbiota. By focusing on the modulation of the gut microbiota and fully accounting for the clinical realities of the NICU, these strategies offer novel, multifaceted avenues with promising translational potential for the precise intervention of post-aSAH cognitive impairment (Table 3).

**Table 3:** Clinical intervention strategies.

Intervention strategy	Potential mechanisms	Clinical phase	Considerations
Dietary interventions (beneficial metabolites)	Directly increases the intake of beneficial metabolites (e.g., SCFAs, TUDCA) to improve cognitive deficits.	Acute phase	Given that patients in acute and critical phases frequently present with coma or dysphagia in clinical practice, conventional dietary interventions could be optimized into enteral nutrition formulations.
Probiotic supplementation (e.g., *Bifidobacterium*)	Modulates the abundance of gut microbiota and regulates specific microbial metabolites (such as LPS, butyrate, and valerate) to ameliorate cognitive impairment.	Chronic recovery phase	Direct administration during the acute phase carries a potential risk of inducing gut-derived bacteremia due to compromised intestinal barriers.
FMT	Directly restores microbial homeostasis, normalizes beneficial metabolites, and enhances synaptic plasticity.	Chronic recovery phase	Impractical in the NICU due to prophylactic/therapeutic administration of broad-spectrum antibiotics and host immunosuppression.
Pharmacological modulation (e.g., TGR5 agonists)	Mimics the neuroprotective effects of bile acids, activating the cAMP/PKA pathway to inhibit NLRP3 inflammasome-mediated neuronal pyroptosis.	Acute phase	Represents “metabolite replacement therapy” that safely circumvents bacterial colonization hurdles during acute vulnerability.
Neuromodulation (e.g., taVNS)	Reactivates parasympathetic tone via the CAP, downregulating the release of intestinal pro-inflammatory cytokines.	Acute and chronic phases	Requires specific non-invasive devices; clinical efficacy may depend on the severity of the primary central autonomic network damage.

To systematically translate these theoretical strategies into clinical practice and address the aforementioned translational barriers, we propose a concrete, actionable framework for future clinical trial designs targeting the microbiota-gut-brain axis in aSAH: (1) Patient selection: Target aSAH survivors with cognitive deficits, strictly excluding those with pre-existing gastrointestinal disorders or active infections requiring prolonged empirical broad-spectrum antibiotics. (2) Phase-stratified interventions: Acute-phase protocols should prioritize “metabolite replacement” (e.g., SCFAs or TGR5 agonists) or neuromodulation (taVNS) to bypass NICU antibiotic barriers. Chronic-phase designs (>30 days post-hemorrhage) should focus on microbiome reconstitution (e.g., FMT or precision probiotics). (3) Longitudinal cognitive endpoints: Utilize standardized, multi-domain neuropsychological batteries at predefined intervals to precisely map recovery trajectories in memory and executive function. (4) Biomarker monitoring: Integrate multi-omics profiling (metagenomics and untargeted metabolomics) using longitudinal fecal and plasma sampling at critical pathological time points. (5) Cohort design: Transition from initial discovery cohorts to adequately powered, multi-center longitudinal validation trials to establish temporal causality, rule out reverse causation, and ensure translational viability for precision cognitive rehabilitation.

## 6 Limitations and unresolved questions

Despite the compelling theoretical framework linking the microbiota-gut-brain axis to post-aSAH cognitive impairment, several critical limitations in the current literature must be candidly acknowledged. Primarily, current research still mainly focuses on the influence of gut microbiota on the formation and rupture of intracranial aneurysms, leaving a significant gap in specific studies regarding the modulatory role of gut microbiota and its metabolites in post-aSAH cognitive impairment (Shikata et al., 2019; Kawabata et al., 2022; Sun et al., 2024; Xu et al., 2024; Csecsei et al., 2025).

A primary methodological hurdle stems from the profound confounding effects of iatrogenic factors in the specific clinical environment of the NICU. Current clinical cohort studies investigating the gut microbiota following aSAH predominantly focus on the acute phase, during which patients typically undergo highly invasive systemic treatments. The routine prophylactic or therapeutic administration of broad-spectrum antibiotics, standardized enteral nutrition formulations, proton pump inhibitors, sedatives and analgesics, alongside the risk of nosocomial infections, substantially reshape the host microbiome (Dickson, 2016; Oami et al., 2025). Currently, it remains nearly impossible to definitively distinguish aSAH disease-specific microbial signatures from treatment-related iatrogenic dysbiosis.

Compounding these clinical challenges is the observational nature of existing human data. While experimental animal models provide evidence for causal mechanisms, human studies remain predominantly cross-sectional and correlative. This cross-sectional nature fundamentally precludes the establishment of temporal precedence between gut dysbiosis and cognitive impairment. Consequently, reverse causation remains a highly plausible alternative explanation: severe early brain injury and subsequent cognitive or motor deficits can profoundly alter a patient’s dietary intake, physical activity, and toileting patterns. These alterations in turn secondarily precipitate microbiome shifts, rather than the dysbiosis acting as the exclusive etiology for the cognitive decline.

Beyond the obstacles of establishing strict causality, the true effect size of microbiota contributions to cognitive decline in aSAH patients remains largely undetermined. This knowledge gap is further compounded by a critical absence of extensive longitudinal follow-up data tracking microbiome reconstitution during the chronic recovery phase. Furthermore, there is a likely publication bias characterized by a lack of published negative findings in microbiome-brain axis research. Addressing these unresolved questions through rigorous, multi-center longitudinal designs that account for NICU confounders is an absolute prerequisite for advancing this field from observational phenomena toward genuine clinical translation.

## 7 Conclusion and future perspectives

**Figure 3:**
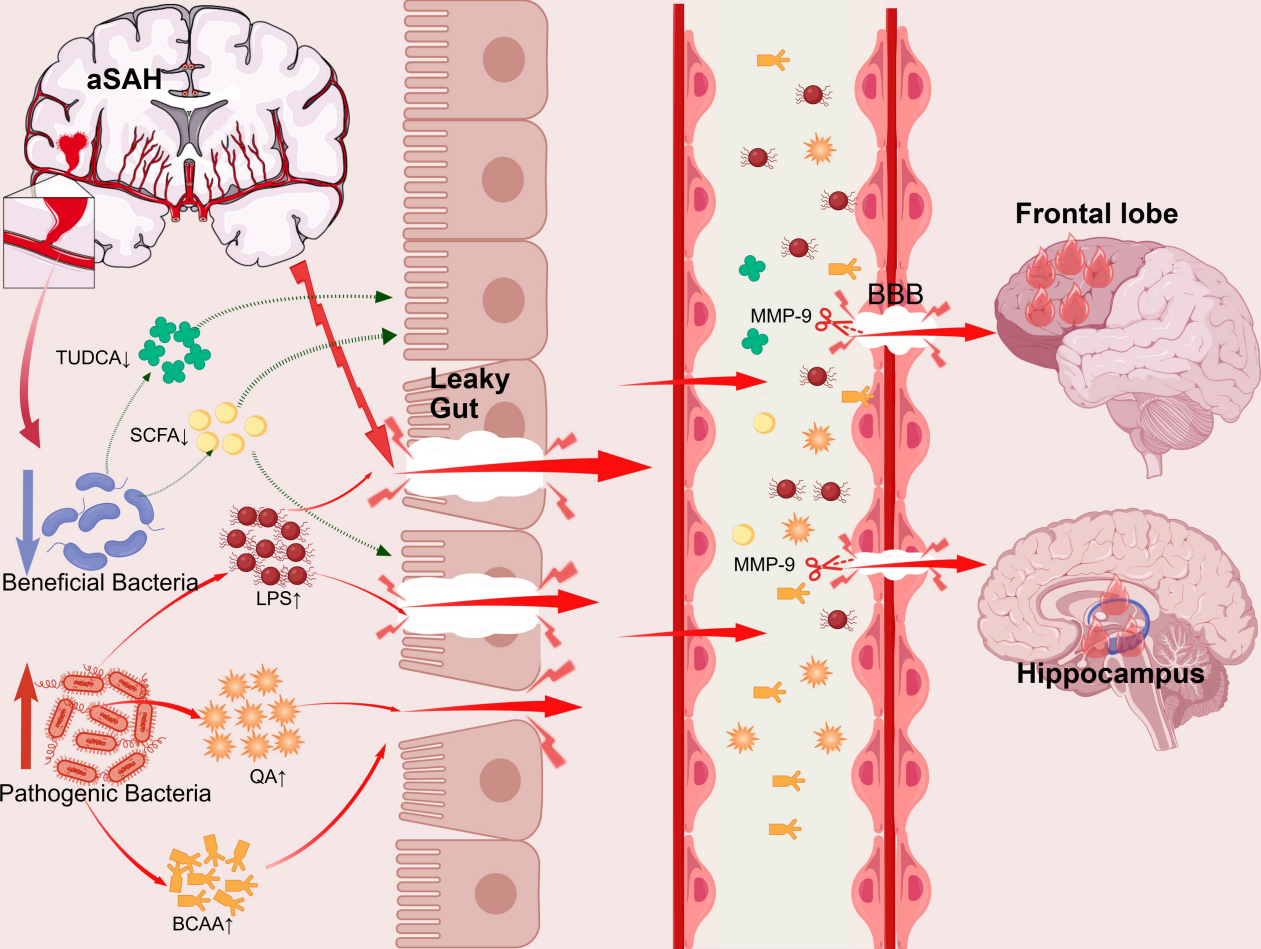
A proposed gut-brain axis model for post-aSAH cognitive impairment. Following aSAH, intrinsic stress conditions, coupled with gut microbiota dysbiosis, manifest as the depletion of beneficial metabolites and the accumulation of deleterious ones. These changes collectively induce intestinal barrier injury and facilitate the translocation of microbial metabolites into the systemic circulation. Furthermore, upregulated expression of MMP-9 promotes the degradation of tight junction proteins, thereby disrupting the integrity of the BBB. This heightened permeability subsequently facilitates the infiltration of gut-microbiome-derived metabolites into the brain parenchyma, ultimately contributing to the progression of cognitive impairment through multifarious mechanisms. Created with BioGDP.com (Jiang et al., 2025).

Long-term cognitive impairment following aSAH presents a profound clinical challenge that severely impedes patients’ reintegration into society. Current mechanistic investigations, however, remain largely confined to the primary central nervous system damage resulting directly from the aneurysm rupture. In this study, we innovatively propose a “gut-derived secondary hit” hypothesis, systematically elucidating how secondary gut microbiota dysbiosis, acting as a consequence of post-aSAH central stress, functions as an active potential modulator that may be associated with long-term cognitive deficits via the microbiota-gut-brain axis (Figure 3). This paradigm shift provides a robust foundation to bridge the theoretical gap between impaired neural remodeling following aSAH and the potential for gut-targeted therapeutic interventions.

We systematically review the clinical manifestations and underlying pathological mechanisms of cognitive impairment following aSAH, delineating the observed compositional shifts in the gut microbiota associated with the disease, and critically examining the potential mechanisms by which the gut microbiota and its associated metabolites may participate in cognitive impairment after aSAH via various pathways such as immune-inflammatory responses, neuroendocrine signaling, neural circuits, oxidative stress, cellular apoptosis, and disruption of the BBB. Additionally, this review summarizes targeted interventional strategies directed at the gut microbiota, including probiotics, dietary modifications, FMT, drug regulation, and neuromodulation, thereby providing novel therapeutic avenues for the prevention and management of aSAH-induced cognitive impairment.

Moving forward, to address the aforementioned limitations, future research must prioritize the integration of clinical interventions with multi-omics technologies, striving to advance from observational phenomena toward rigorous mechanistic validation and clinical translation. Specifically, overcoming the profound confounding effects of broad-spectrum antibiotics and enteral nutrition requires sophisticated statistical frameworks. Future studies must employ advanced dimensionality reduction and penalized regression models, such as Least Absolute Shrinkage and Selection Operator (LASSO) regression and propensity score matching, to rigorously adjust for high-dimensional clinical covariates and isolate true disease-specific microbial signatures. Furthermore, it is essential to transition from initial multi-omics discovery cohorts toward adequately powered, multi-center longitudinal validation cohorts. This longitudinal approach is the only way to establish temporal precedence and definitively rule out reverse causation.

By conducting longitudinal fecal and plasma sampling at critical pathological time points, such as the acute hemorrhagic phase, the delayed cerebral ischemia phase, and the chronic recovery phase at 6 months post-discharge, and coupling these with integrated metagenomic and untargeted metabolomic profiling, this approach holds the promise of successfully identifying and establishing pivotal gut-derived candidate biomarkers (Puig-Castellví et al., 2023; Ona et al., 2025). For instance, monitoring abnormally elevated plasma levels of BCAAs, alongside significantly depleted fecal SCFAs (e.g., butyrate) and secondary BAs (e.g., TUDCA), could serve as crucial indicators for assessing the severity of microbiota-gut-brain axis dysfunction and predicting cognitive outcomes in patients. Utilizing logistic regression and evaluating diagnostic model performance via ROC (Receiver Operating Characteristic) curve analysis will facilitate the construction of precise prognostic models. Furthermore, given the inevitable reliance on broad-spectrum antibiotics within the NICU, the acute-phase application of live biotherapeutics, such as probiotics or FMT, encounters formidable translational barriers. Consequently, future therapeutic strategies should pivot toward “metabolite replacement therapy.” By directly administering neuroprotective metabolites (e.g., SCFAs or TGR5 receptor agonists) during the acute phase, clinicians can effectively circumvent the obstacles associated with bacterial colonization. This innovative strategy effectively bypasses microbiome engraftment hurdles to directly target compromised neural circuits. When coupled with the subsequent reconstruction of the gut microecology during the chronic recovery phase, this approach will pioneer a safer and more viable avenue for the cognitive rehabilitation and precision management of patients with aSAH.
